# A Case of Symmetrical Gangrene (Raynaud’s Disease), with Some Unusual Features in Its Etiology

**Published:** 1896-02

**Authors:** M. B. Hutchins

**Affiliations:** Atlanta, Ga., Clinical Lecturer on Dermatology and Syphilis, Demonstrator of Anatomy and Histology, Atlanta Medical College


### A CASE OF SYMMETRICAL GANGRENE (RAYNAUD'S DISEASE), WITH SOME UNUSUAL FEATURES IN ITS ETIOLOGY.

BY M. B. HUTCHINS, M.D., ATLANTA, GA.,

Clinical Lecturer on Dermatology and Syphilis, Demonstrator of Anatomy and Histology, Atlanta Medical College.

The case to be described came under my care nearly a year ago, and the report is copied almost literally from my notes. A history of the characteristic early symptoms of Raynaud's disease was not obtained, but the clinical features of the disease in its later stages justified the diagnosis.

A typical case of symmetrical gangrene usually begins with periodic attacks of syncope followed by asphyxia of the affected parts. The common site of the disease is the fingers of each hand, the toes of the feet, both ears; and rarely some other portion of the body. The condition has been ascribed to nervous action, central or peripheral, or, as usually agreed now, to arterial sclerosis. Varicose veins alone do not appear to have been credited with any effect in its causation, but if the varicosity is sufficiently extensive and reaches to the peripheral vessels, it would seem reasonable to suppose that the stagnation in the veins might act upon the tissues directly behind the origin of the venous radicles.

It is possible that there was a coexisting arterial sclerosis or contraction which was not made out in the examination of the case and which might not have been discovered without a *post-mortem* examination.

Following are notes of the case:

February 25, 1895.—W. T., age fifty. Farmer.

Diagnosis (first) *Venæ Varicosæ et Sequelæ.* (The "sequelæ" being the pompholyx-like lesions on the toes.)

"Formerly stood on feet a great deal. Trouble with the legs for about seven years. Condition present on toes since first of December, 1894. At present, the skin and subcutaneous tissues of the legs, dorsal surfaces and sides of the feet show numerous tortuous, dilated veins, with their walls here and there thickened into hard 'lumps' beneath the skin. Large patches of pigmentation mark the site of old ulcers on the leg and ankle.

On the plantar surface of the end of several toes are large, flat, bullæ, their contents giving them a blackish purple color. (Distal part of soles rather thickened.) Blebs which have been broken have healed, leaving callosities (from excess of horny formation). Toes benumbed when cold.

The general health is good (apparently). Formerly followed the occupation of a coppersmith. Treatment:

| | | |
|---|---|---|
| Bandage, toes to knee. | ℞ Resorcin pulverat....... gr. xv.<br>Acid. salicyl.................gr. xx.<br>Talc venenat...............℥ i.<br>M. Sig: Keep well on lesions. | Open blebs with a clean needle, express contents. |

March 2.—Toes of right foot improved. Those of left *in statu quo*.

March 5.—As before. Treatment continued, with use of foot-baths of hot salt water at night. A three per cent. aqueous solution of ichthyol used to soften the dressings and cleanse the lesions. Begins to wear elastic stockings covering foot and leg from the metatarsels to just below the knee."

On March 18th, the conclusion was reached that both the diagnosis and the treatment were inadequate.

At that time the following notes were made:

"Left foot: On the inner side of the plantar surface of the great toe a discoid, half-dime size, hard crust covering livid, granulation-like tissue. On the plantar surface, end of the second toe, a dime-sized similar lesion, covered with a hard, adherent crust. (These "crusts" were largely composed of fibrous tissue and epidermis.) Upon removal of them, large livid granulations were exposed—forming a ring around the center where the periosteal covering at the end of the phalanx was exposed. On the third toe, a similar, but smaller, lesion.

After thorough removal of the "crusts" with scissors, the granular tissue was cauterized with fuming nitric acid, and the surfaces dressed with pulverized iodoform.

March 20th, no change in left great toe, end of bone exposed in second, and nearly so in third. Unhealthy, livid, granulation tissue persists. Surgical removal of the diseased toes was advised, and declined.

On the inner side of the right great toe, plantar surface, a one-half by three-fourths of an inch, blackish, soft, adherent mass of necrotic tissue, loose at its edges, continuous in the middle with the fascia, with a limiting ring of fairly healthy looking granulations under its border. A four per cent. solution of nitrate of silver was applied and the iodoform dressings continued. The skin of the diseased toes all somewhat livid in color. Beneath the second and third toes of the right foot dark discolorations, the probable site of former lesions. A healed one on the left little toe shows a normal color with callous covering." The elastic stocking not extending to the base of the toes, as it should, it was necessary to supplement it with a bandage.

The suggestion of amputation ended my connection with the case, the affected parts separated spontaneously, and a few months ago the papers announced the rather sudden death of the patient at his home in a neighboring village.

311–312 *Fitten Building.*

